# Evaluation of Expression of Cytochrome P450 Aromatase and Inflammatory, Oxidative, and Apoptotic Markers in Testicular Tissue of Obese Rats (Pre)Treated with Garlic Powder

**DOI:** 10.1155/2023/4858274

**Published:** 2023-01-04

**Authors:** Baicheng Li, Jia Li, Abolfazl Akbari, Payam Baziyar, Shuwen Hu

**Affiliations:** ^1^Department of Pharmacy, Shaanxi Provincial Hospital of Chinese Medicine, Xi'an 710003, China; ^2^Department of Endocrine Genetics and Metabolism, Xi'an Children's Hospital, Xi'an 710003, China; ^3^Department of Physiology, School of Veterinary Medicine, Shiraz University, Shiraz, Iran; ^4^Department of Molecular and Cell Biology, Faculty of Basic Science, University of Mazandaran, Babolsar, Iran

## Abstract

Today, adolescent obesity is recognized as an epidemic and a cause of reproductive disorders. Decreased testosterone levels occur due to functional defects in the hypothalamus-pituitary axis, excessive activity of cytochrome P450 aromatase enzyme, and testicular dysfunction in these people. Oxidative damage, inflammation, and apoptosis are also the main mechanisms of testicular damage during obesity. The use of herbal products such as garlic can improve this disorder due to its anti-inflammatory and antioxidant properties. Therefore, the aim of this study is to investigate the effect of pretreatment and treatment of garlic powder on the expression of cytochrome P450 aromatase enzyme and the expression of genes involved in testosterone synthesis, inflammation, oxidative damage, apoptosis in testicular tissue, and metabolic function of liver tissue in young male obese rats. Eighty male Wistar rats were divided into the controlled and treated groups. Serum levels of lipid, glucose, and insulin as metabolic factors were measured along with the testicular antioxidant and inflammation markers. The expression of Bcl2, Bax, and caspase-3 along with NF-*κ*B, SREBP-1c, CPT-1beta, Nrf-2, CD36, FAS, CYP19A1, P450scc, StAR, 17*β*HSD, PPAR*α*, and aromatase (CYP19, P450arom) was also measured. Testicular histological evaluation and spermatogenic process was also performed. The results showed that oxidative, inflammatory, and metabolic factors significantly increased in obese rats. The testicular expression of aromatase, NF-*κ*B, Bax, and caspase 3 increased and Nrf2 expression decreased in obese rats, while (pre) treatment with garlic powder significantly decreased the expression of these genes in obese rats. These results were also confirmed by the findings of the histological evaluation and sperm analysis. It can be concluded that garlic powder could improve reproductive dysfunction in obese rats.

## 1. Introduction

Obesity refers to abnormal and considerable body fat accumulation with a harmful effect on health. According to the definition of the World Health Organization (WHO), obesity is when the body mass index (BMI) is higher than 30 kg/m^2^. The number of adults over 18 years of age affected by obesity and its complications exceeds 1.9 billion worldwide [1]. Obesity is associated with many disorders, including diabetes mellitus, hypertension, coronary heart disease, and infertility disorders [2]. Studies have shown that the increase in obesity rates has a relationship with the rising rates of male infertility [3], and it is responsible for 45%–50% of infertility in couples [4]. Obesity can change testosterone, LH, and FSH levels, functional parameters of sperm, induce oxidative stress, and inflammation and facilitates epigenetic modifications that are transmitted to offspring as well [3, 5]. Obesity-related hypogonadism and its effects on spermatogenesis are other negative effects associated with obesity-induced infertility [3]. Oxidative stress related to cellular damage caused by oxygen, superoxide anion, and superoxide is generally known as reactive oxygen species (ROS) [6, 7]. The testicular tissues and spermatozoa are very vulnerable to ROS attack and lipid peroxidation [8–10]. ROS produced by free fatty acids attack the cell membrane and cause damage to DNA and DNA fragmentation, which activates signaling pathways inducing testicular cell apoptosis [11–13]. The findings of the studies showed that ROS stimulated lipid peroxidation in the germ cell membrane and induced apoptosis in these cells [7, 12, 13]. Moreover, the improper level of some vitamins and elements [14, 15] and adipokines such as resistin [16] significantly plays a key role in metabolic and inflammatory responses such as insulin/leptin signaling in obese patients. In addition, the NF-*κ*B pathway is activated and increased the release of proinflammatory factors like IL-1*β*, IL-6, and TNF-*α*, which can induce cell apoptosis [17, 18]. This process can play a significant role in infertility due to local inflammation, especially in the genital area [19]. In addition, obesity-related inflammation leads to increased expression of the CYP19A1 gene in men and thus leads to an excessive increase in aromatase in obese men, which can be one of the causes of infertility in these people. The aromatase encoded by the CYP19A1 gene is part of the cytochrome P450 superfamily, and it catalyzes the irreversible conversion of testosterone and androstenedione into estrogen, which is a major enzyme in estrogen synthesis [20–22]. Therefore, these interconnected pathways and mediators involved in them are very appropriate therapeutic goals to improve obesity and its infertility complications. Increasing physical activity through regular exercise and lifestyle changes through diet correction and the use of herbal products are very simple strategies that are highly regarded [23–29].

The medicinal plants and the products are preferred by many, when given the low cost, availability, and low side effects [30, 31]. Garlic (*Allium sativum* L.) is an annual plant that uses as a medicinal plant and spice and has been used for centuries to protect the body against infections [32]. Recently, several studies have reported that garlic has notable biological functions such as anticancer, antioxidant, cardiovascular protective, antidiabetic, anti-inflammatory, immunomodulatory, antibacterial, and antiobesity [33–37]. Moreover, one of the major garlic medical values is its antiatherogenic and lipid-lowering effects on animals and humans. Using garlic has a significant lowering effect on plasma lipid mostly total cholesterol and low-density protein (LDL) cholesterol in man. Adding garlic to diet decreases the hepatic activities of cholesterogenic and lipogenic enzymes like 3 hydroxy-3-methylglutaryl-CoA (HMG CoA) reductase, glucose-6-phosphate dehydrogenase, and malic enzyme. In addition, a recent study showed that garlic could improve sperm parameters and testosterone production in rats exposed to furan [38]. El-Akabawy and El-Sherif [38] also reported that garlic coadministration improved testosterone and considerably decreased the furan-induced oxidative, apoptotic, and histopathological changes in the adult rat testis. Moreover, it was reported that garlic can improve reproductive function in animal model by inhibiting oxidative damage [39]. Sheweita et al. [39] showed that garlic (800 mg/kg), selenium (1 mg/kg body weight), and/or their combination for up to three months (three times/week) can reduce the toxic effects of tramadol in adult male rabbits [39]. Lai et al. [40] also reported that “Garlic essential oil can protect against obesity-triggered nonalcoholic fatty liver disease through modulation of lipid metabolism and oxidative stress” [40]. Although some studies have examined the role of garlic in modulating metabolic parameters and some reproductive characteristics its role in altering histological characteristics, the molecular mechanisms involved in testosterone synthesis, pituitary-gonadal axis, oxidative damage, inflammation, and apoptosis in obese rats need to be studied. Thus, the present study is an attempt to examine the expression of aromatase cytochrome P450 enzyme and the antioxidant, anti-inflammatory, and antiapoptotic effects of garlic powder on reproductive defects due to obesity in young male rats.

## 2. Materials and Methods

### 2.1. Material Plant

Garlic (*Allium sativum*) was procured from a local market by a botanist, and it was identified and checked on https://www.theplantlist.org. Normal saline was used for dose preparation.

### 2.2. Total Phenolic Content and the Total Antioxidant Activity

To determine the total phenolic content (TPC) and the total antioxidant capacity, Folin–Ciocalteu spectrophotometric method [41] and the scavenging activity of 1,1-diphenyl- 2-picrylhydrazyl (DPPH) radical [42] were used, respectively.

### 2.3. Animals

Eighty male Wistar rats (230 ± 20 g, 6–8 weeks) were kept based on the standard conditions from animals in the lab (relative humidity 38%, 22°C, and 12/12 hours of light/dark cycle). The subjects were allowed free access to the standard diet and water. The “Guidelines for the Care and Use of Laboratory Animals” was used for animal care and for observing ethics. The Ethics Commission with ethical number EC20210006558 approved the study.

### 2.4. Study Design

To cause obesity, the subjects were fed a high-fat diet (HFD) for 12 weeks and it was checked using the HOMA-IR evaluation test [3]. After one-week of adaptation, the subjects were assigned randomly to six controlled and diabetic groups as explained in the following:

  Group 1: control rats were provided with distilled water orally as a vehicle using gavage (12 weeks)
  Group 2: obese rats were provided with distilled water orally as a vehicle using gavage (12 weeks)
  Group 3: healthy rats were orally fed with garlic powder (50 mg/kg/day) using gavage (12 weeks)
  Group 4: healthy rats were orally fed with garlic powder (100 mg/kg/day) using gavage (12 weeks)
  Group 5: obese rats were orally fed with garlic powder (50 mg/kg/day) using gavage (12 weeks)
  Group 6: obese rats were orally fed with garlic powder (100 mg/kg/day) using gavage (12 weeks)
  Group 7: animals were orally protected by gavage for 12 weeks during induction of obesity with garlic powder (50 mg/kg/day)
  Group 8: animals were orally protected by gavage for 12 weeks during induction of obesity with garlic powder (100 mg/kg/day)

### 2.5. Sampling, Tissue Preparation, and Measurement of Biochemical Parameters

Following the final treatment session fasting for one night, blood samples were taken through cardiac puncture. After centrifuging (3000 g, 10 min), the collected serums were tested to determine insulin, lipid profile, and glucose. The parameters except for insulin were measured using Hitachi 912. To determine the serum level of insulin, the ELISA method was utilized along with a rat-specific kit (Linco Research Inc., St. Charles, MO, USA). Following the sampling, the testis tissue was separated and weighed. Testicular and serum testosterone levels were measured by ELISA using an existing commercial kit (CSB-E05100r kit, CUSABIO, Wuhan, China). The CAT activity of tissue was examined in terms of the degradation rate of hydrogen peroxide using CAT read at 230 nm [43]. The total amount of SOD activity in testicular tissue was measured following Misra and Fridovich, [44]. The activity of GSH-Px was measured using NADPH oxidation rate at 340 nm using hydrogen peroxide as the substrate in a coupled assay with glutathione reductase [45]. The amount of MDA as a byproduct of lipid peroxidation was determined in testicular tissue following Ohkawa et al. [46]. The testes levels of IL-1*β* and TNF-*α* were determined using ELISA by a rat-specific kit (Linco Research Inc., St. Charles, MO, USA). The concentration of protein in testes was measured using Bradford and bovine serum albumin was used as the standard [47].

### 2.6. Molecular Measurement

To assess reproductive damage caused by obesity, testicular expression of genes that had a role in the production of testosterone and P450Aromatase was measured along with oxidative stress, apoptosis, and inflammation. Total RNA extract kit was used to extract total RNA (Jena Bioscience, Germany). Primer Script reverse transcriptase was used to synthesize complementary DNA (cDNA) from RNA samples (TaKaRa, Japan). Here, the primers of target genes were selected following the references [42, 48].  Table 1 lists the sequence of the primers. Real Q Plus 2x Master Mix Green (Amplicon, Denmark) was used in real-time PRC device (Applied Biosystem, USA) to perform real-time PCR. Normalization of the target genes expression was done compared to *β*-actin expression. In addition, the method 2^−ΔΔCT^ was used to determine the relative expression level.

### 2.7. Histological Examination

The left testicles were fixed in 4% formaldehyde solution. After at least a week, they were embedded in paraffin and incisions approximately 5 *μ*m thick were made using a microtome. After staining, using hematoxylin and eosin (H&E), the evaluation of the prepared cross-sections was done based on the study of Memudu et al. [49]. The state of spermatogenesis in the seminiferous tubules was also evaluated by Jansen's score [50]. 50 spermatogenic tubes were examined at each stage and were given a score of 1–10 based on the following criteria: 10 means complete spermatogenesis and perfect tubules; 9 means many spermatozoa present but disorganized spermatogenesis; 8 means only a few spermatozoa are present; 7 means no spermatozoa but many spermatids present; 6 means only a few spermatids are present; 5 means no spermatozoa or spermatids are present but many spermatocytes are present; 4 means only a few spermatocytes are present; 3 means only spermatogonia present; 2 means no germ cells are present; and 1 means neither germ cells nor Sertoli cells are present.

### 2.8. Statistical Analysis

The data were recorded in SPSS 18. One-way analysis of variance (ANOVA) and Tukey post hoc test were used to compare the differences between the groups. The results were presented as the means ± standard deviation (SD). *p* < 0.05 was considered as the minimum statistically significant level.

## 3. Results

**Figure 1:**
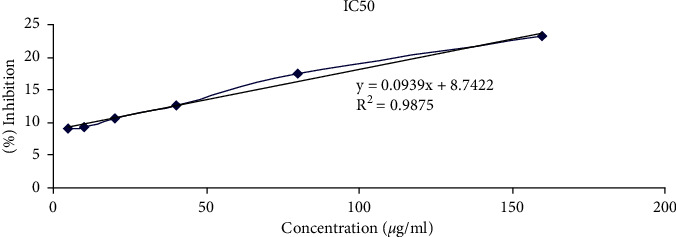
DPPH radical scavenging activity of garlic powder.

Table 1 and Figure 1 represent the DPPH results. As indicated, the garlic powder demonstrated a good activity of scavenging with DPPH (IC50 = 439.3 *μ*g/mL). In addition, total phenol content was determined using the calibration curve of the equation and reported as *μ*g of gallic acid equivalents per mg of sample (*μ*g of GAE/mg of the sample) equal to 6863.59 *μ*g GAE/mg sample.

### 3.1. Body Weight and Food Intake

**Figure 2:**
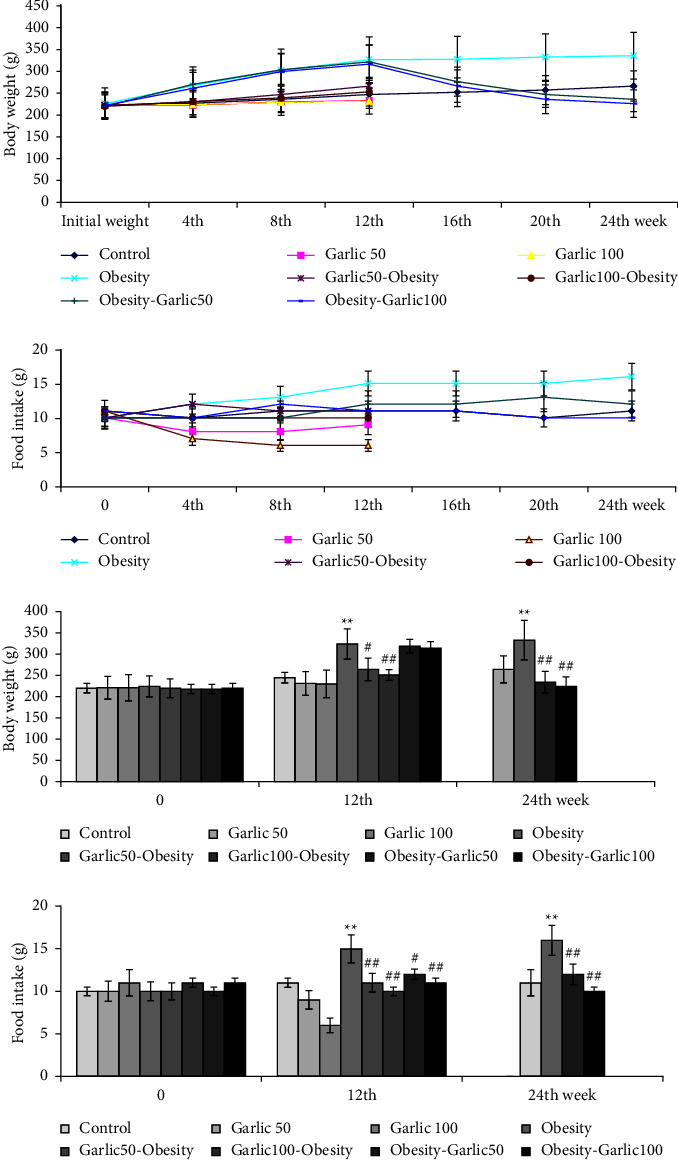
The mean ± SD levels of food intake and body weight (g) in controlled and (pre)treated rats. The star (^∗∗^: *p* < 0.01) represent a significant difference with the control group. Square (^#^: *p* < 0.05, ^##^: *p* < 0.01) shows a significant difference with obese rats.

The results showed that consuming a high-fat diet for 12 weeks significantly improved food intake and weight gain (*p* < 0.05, Figure 2). However, the consumption of garlic powder in different doses, both therapeutically and protectively, was able to significantly reduce body weight in obese and overweight rats (*p* < 0.05, Figure 2). Moreover, food intake in treatment and pretreatment groups with garlic was significantly reduced (*p* < 0.05, Figure 2). This indicates the role of garlic in controlling appetite. In this study, we measured the weight of liver and testicular tissues to assess tissue index (*p* < 0.05, Table 2). The results showed that the weight of the liver tissue and testicular tissue in rats receiving high-fat diet changed significantly in comparison with the control group. Moreover, using different doses of garlic powder for pretreatment and treatment can improve them (*p* < 0.05, Table 2).

### 3.2. Status of Glucose, Insulin, and HOMA-IR in Different Groups

In this study, in order to evaluate the obesity status and the effect of treatment and pretreatment with different doses of garlic powder, the serum levels of insulin, glucose, and the HOMA-IR index were determined. To examine resistance to insulin in nonobese and obese rats under treatment and pretreatment of diverse doses of garlic powder, the HOMA-IR index was utilized. The results are presented in Table 3. In comparison to other groups, the mean ± SD of the fasting blood glucose (mg/dl) level, insulin (*μ*U/L) level, and HOMA-IR index significantly increased in the obesity group (*p* < 0.05, Table 3). On the other hand, pretreatment and treatment with diverse doses of garlic powder significantly enhance these parameters (*p* < 0.05, Table 3).

### 3.3. Treatment and Pretreatment with Different Doses of Garlic Powder Could Modulate Glucose and Lipid Metabolism by Targeting the Genes Expression Taking Part in the Oxidation and Lipogenesis of Fatty Acid

**Figure 3:**
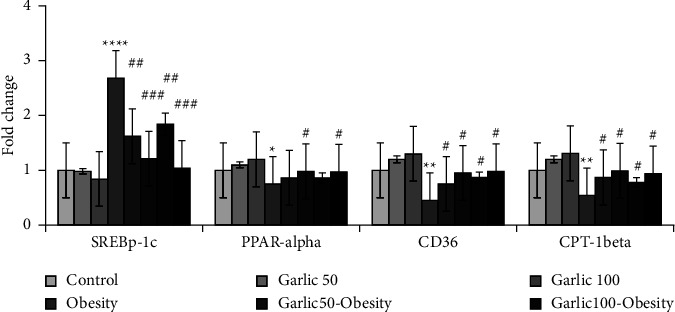
The mean ± SD mRNA fold change of SREBP-1c, PPAR*α*, and CD36, CPT-1, in liver tissue of the different groups. The star (^∗^: *p* < 0.05, ^∗∗^: *p* < 0.01, ^∗∗∗∗^: *p* < 0.0001) represent a significant difference with the control group. Square (^#^: *p* < 0.05, ^##^: *p* < 0.01, ^###^: *p* < 0.001) shows a significant difference with obese rats.

In addition to the glucose levels, we measured blood lipid profiles to evaluate the effect of different doses of garlic powder on them. The mean ± SD serum levels of TG, TC, LDL, and glucose were significantly improved by the high-fat diet (*p* < 0.05, Table 4), while treatment and pretreatment with different doses of garlic powder could decrease these metabolic parameters (*p* < 0.05, Table 4). In this study, we also measured the hepatic expression of gene SREBP-1c that has a role in lipogenesis and the expression of genes PPAR*α*, CD36, and CTP-1beta, which have a role in the fatty acid metabolism of fats in different groups (Figure 3). The PPAR*α*, CD36, and CTP-1beta expression significantly decreased and SREBP-1c expression increased by HFD (*p* < 0.05, Figure 3). In addition, treatment and pretreatment with different doses of garlic could modulate these results (*p* < 0.05, Figure 3).

### 3.4. Garlic Powder Could Inhibit Oxidative Damage Caused by Obesity in Testicular Tissue

**Figure 4:**
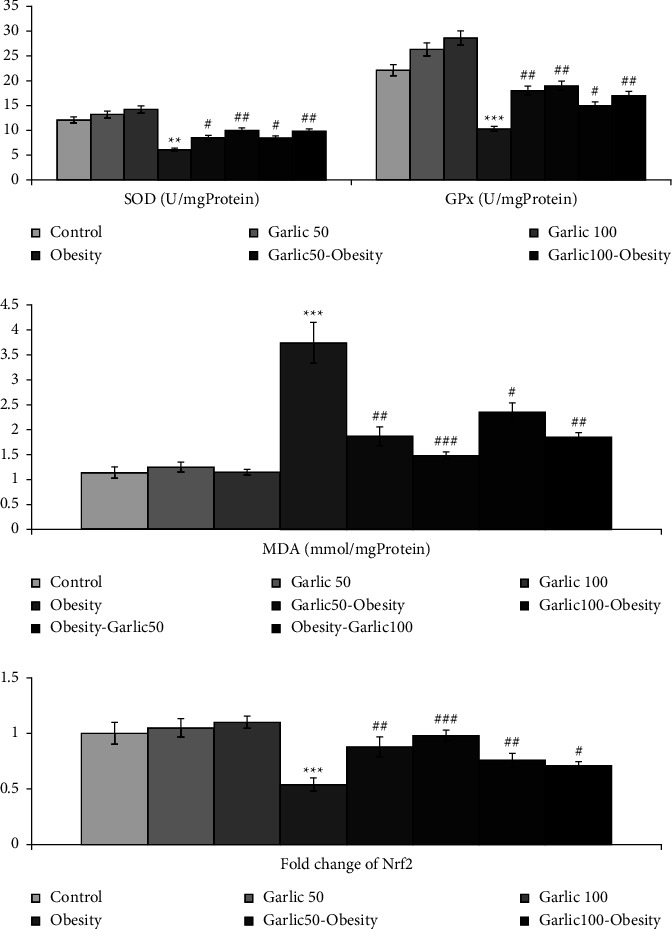
The mean ± SD of Nrf2 expression, SOD and GPx activity, and MDA level in different groups. The star (^∗∗^: *p* < 0.01, ^∗∗∗^: *p* < 0.001) represents a significant difference with the control group. Square (^#^: *p* < 0.05, ^##^: *p* < 0.01, ^###^: *p* < 0.001) shows a significant difference with obese rats.

Consumption of high-fat diet could reduce the activity of enzymes SOD and GPx and increase the amount of MDA in testicular tissue (*p* < 0.001, Figure 4). However, treatment and pretreatment with different doses of garlic powder could inhibit oxidative damage by increasing the expression of Nrf2 and the activity of SOD and GPx (*p* < 0.05, Figure 4). Nrf2 was measured as one of the transcription factors controlling cell redox status in order to monitor the antioxidant enzymes activity. Expression of this transcription factor in testicular tissue in response to high-fat diet demonstrated a significant decline (*p* < 0.05, Figure 4). In addition, treatment and pretreatment with different garlic powder doses could significantly increase its level compared to the obesity group without any intervention (*p* < 0.05, Figure 4).

### 3.5. Garlic Powder Could Inhibit Inflammation by Decreasing the Activity of TNF-*α* and NF-*κ*B Expression

**Figure 5:**
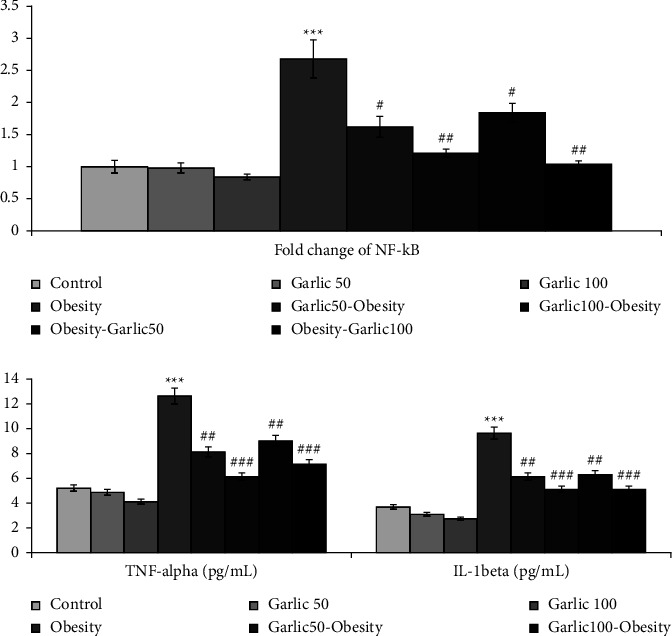
The mean ± SD of TNF-*α* and IL-1beta level and mRNA fold change of NF-*κ*B in testicular tissue of the different groups. The star (^∗∗∗^: *p* < 0.001) represent a significant difference with the control group. Square (^#^: *p* < 0.05, ^##^: *p* < 0.01, ^###^: *p* < 0.001) shows a significant difference with obese rats.

The expression of NF-*κ*B and the activity of inflammatory factors IL-1*β* and TNF-*α* significantly increased in testicular tissue with high-fat diet (*p* < 0.001, Figure 5). Pretreatment and treatment with garlic powder (50 and 100 mg/kg) could significantly inhibit the inflammatory status by reducing expression of NF-*κ*B and activity of TNF-*α* and IL-1*β* (*p* < 0.05, Figure 5).

### 3.6. Garlic Powder Could Inhibit Obesity-Induced Apoptosis in Testicular Tissue

**Figure 6:**
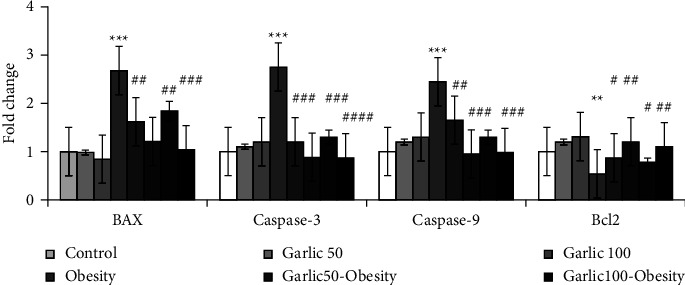
The mean ± SD mRNA fold change of genes involved in apoptosis. The star (^*∗∗*^: *p* < 0.01, ^*∗∗∗*^: *p* < 0.001) represent a significant difference with the control group. Square (^#^: *p* < 0.05, ^##^: *p* < 0.01, ^###^: *p* < 0.001, ^####^: *p* < 0.0001) shows a significant difference with obese rats.

The expression of BAX, Cas-3, and -9 genes significantly increased and the expression of Bcl2 gene decreased in testicular tissue in response to the consumption of high-fat diet (*p* < 0.001, Figure 6). Pretreatment and treatment with garlic powder could significantly inhibit the apoptotic events by reducing expression of BAX, Cas-3, and-9 genes and increasing Bcl2 gene expression (*p* < 0.05, Figure 6).

### 3.7. Pituitary-Gonadal Axis Dysfunction in Obese Rats Could Be Improved by Pretreatment and Treatment with Garlic Powder

**Figure 7:**
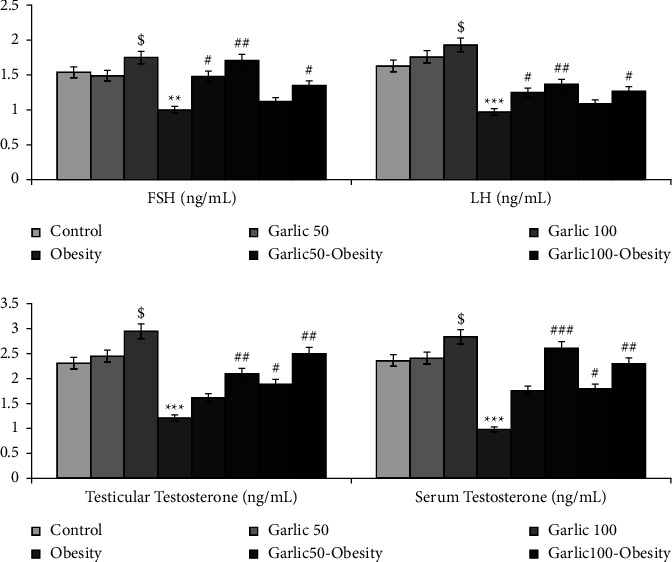
The mean ± SD of level of LH, FSH, and testosterone in the different groups. The dollar ($: *p* < 0.05) and star (^∗∗^: *p* < 0.01, ^∗∗∗^: *p* < 0.001) represent a significant difference with the control group. Square (^#^: *p* < 0.05, ^##^: *p* < 0.01, ^###^: *p* < 0.001) shows a significant difference with obese rats.

In this study, the serum levels of LH, FSH, and testosterone were measured to investigate the effects of obesity and using different garlic powder doses on the pituitary-gonadal axis. The serum levels of LH and FSH were significantly decreased in obese animals compared to the control group. However, pretreatment and treatment with garlic powder could inversely increase the levels of these hormones (*p* < 0.05, Figure 7). Serum testosterone levels also decreased with obesity and showed a significant increase in obese animals with pretreatment and treatment with garlic powder (*p* < 0.05, Figure 7).

### 3.8. Decreased Testosterone Levels in Obese Rats Could Be Improved by Treatment and Pretreatment with Garlic Powder

**Figure 8:**
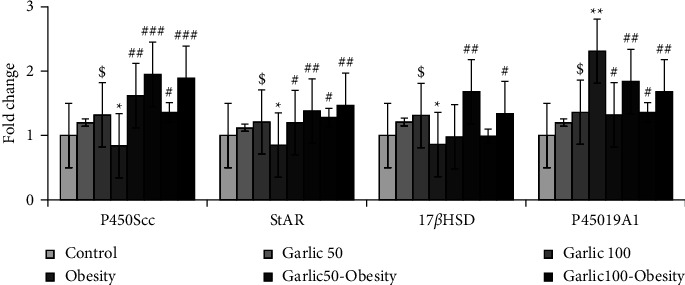
The mean ± SD of genes involved in testosterone synthesis in the different groups. The dollar ($: *p* < 0.05) and star (^∗^: *p* < 0.05, ^∗∗^: *p* < 0.01) represent a significant difference with the control group. Square (^#^: *p* < 0.05, ^##^: *p* < 0.01, ^###^: *p* < 0.001) shows a significant difference with obese rats.

In parallel with the decrease in testosterone levels, the expression of P450Scc, StAR, and 17*β*HSD genes significantly decreased and CYP19A1 expression increased in the obesity group compared to the control group (*p* < 0.05, Figure 8). However, treatment and pretreatment with garlic powder could increase the level of testosterone in serum and testicular tissue by modulating these genes expression (*p* < 0.05, Figure 8).

## 4. Histological Findings

**Figure 9:**
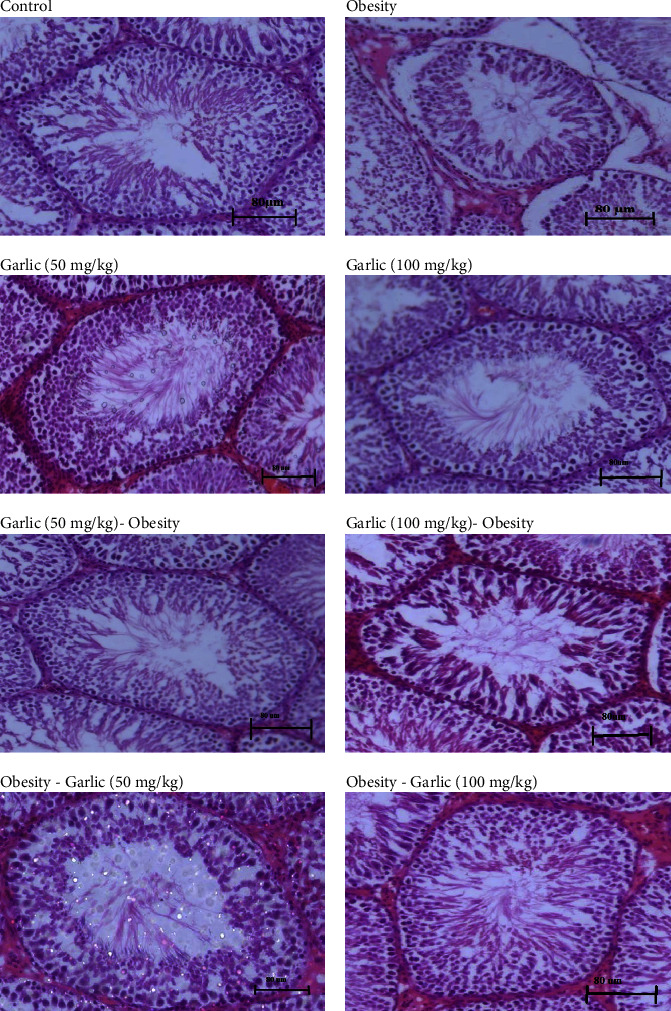
Light microscopic examination of the testicular tissue sections in the control group. The garlic 50 and 100 groups showed normal arrangements and structure for the testis tissue and the normal process of spermatogenesis. Disruption of spermatogenesis and the presence of lesions in testicular tissue were clearly visible in the obese animals. However, these characteristics could be significantly improved by treatment and pretreatment with garlic powder.

In this study, the histological evaluation of the testicular tissue was examined. Destruction of testicular tissue, degeneration of seminiferous tubes, and abnormal spermatogenesis are clearly evident in the testicular tissue of obese rats. However, treatment and pretreatment with different garlic powder doses could exert significant healing effects on the tissue (Figure 9). The number of spermatogonia, spermatocytes, spermatids, Leydig cells, and Sertoli cells was evaluated to examine the process of spermatogenesis (Table 5). Although the number of these cells significantly decreased in the obesity group, treatment with garlic could increase their numbers (*p* < 0.05, Table 5).

## 5. Discussion

Numerous studies have revealed that obesity can be a major development of male infertility and reproductive disorders, caused by the large amounts of free radicals produced by accumulated body fat [1, 51]. Moreover, the chronic inflammatory response patients with obesity triggers immune cells to generate free radicals and leads to increased oxidative stress, which also contributes to cellular oxidative damage and apoptosis accelerates testes damage and dysfunction [24, 52–55]. In addition, there is evidence that leptin exerts a significant role in regulating the male reproductive system. A rise in leptin levels is closely linked to a decrease in testosterone production and increased testicular apoptosis in obese individuals [56, 57]. Therefore, obesity-associated male infertility is associated with increased testicular cytotoxic pathways such as inflammation and apoptosis predominately occurring via a ROS-mediated pathway [10, 25]. In agreement with this evidence, our findings indicated that using a high-fat diet for 90 days causes reproductive and metabolic disorders. It could increase body weight, change the testis index and liver index, and hepatic metabolism of lipids. It also caused reproductive dysfunction by inducing inflammation, oxidative damage, apoptosis in the testicular tissue, decreasing testosterone synthesis, and disturbing of the pituitary-gonadal axis. Some studies in line with our results have also shown that oxidative factors produced during interventions such as varicocele can also cause infertility through changes in the expression of sex hormone receptors and induction of apoptosis [24, 25]. However, our results indicated that pretreatment and treatment with different garlic powder doses had an effective role in controlling body weight, tissue indices, and modulating the mechanisms mentioned in this study.

Weight management strategies based on nutritional and herbal therapies have been highly recommended in recent years [58–61]. In this regard, we investigated garlic, as a protective and therapeutic adjuvant, on body weight, food intake, lipid metabolism, reproductive function, oxidative stress, inflammation, and apoptosis in rats receiving the HFD. Our finding indicated that oral administration of different doses of garlic powder could improve obesity-induced reproductive dysfunction. However, garlic could exert the therapeutic and protective effects through inhibition of obesity-induced (a) inflammation, (b) oxidative damage, (c) in apoptosis testis tissue, (d) enhancement testosterone synthesis by increasing the expression genes of StAR, P450scc, and 17BHSD or decreasing P450aromatase expression, (e) strengthening the activity of the pituitary-gonadal axis, and (f) modulating glucose and lipid metabolism by targeting the expression of genes with a role in lipogenesis and oxidation of fatty acid in the liver tissue.

Consistent with previous studies reported on humans and animals, the findings indicated that garlic could improve the weight of obese animals [62–64]. These researchers indicated that both the therapeutic and protective impacts of garlic are associated with reduced energy intake and body weight [63, 65]. Pretreatment and treatment with garlic could reduce the weight of liver and testicular tissues in rats fed HFD. Moreover, high level of lipid profile (TC, TG, and LDL), glucose, insulin, and HOMA-IR index in HFD rats could decrease in garlic-(pre)treated groups. Increased levels of LDL and TG in obese rats can be due to the decrease in lipoprotein lipase activity [48]. The higher levels of LDL in rats with obesity might be explained by the decrease in LDL receptors, consistent with the previous reports [66–68]. Consistent with our results, it was showed that the administration of garlic extract (250 mg/kg/day) for 60 days decreased the body weight, HOMA-IR, insulin, and glucose in HFD rats. Moreover, ethanolic extract of garlic (100, 250, and 500 mg/kg) could decrease weight gain caused by HFD along with adipose tissue contained in [69]. Pintana et al. [70] also showed that “garlic extract (250 and 500 mg/kg/day) for 28 days enhanced cognitive performance in obese rats” [70]. Garlic acts as an antiobesity agent that neutralizes the impacts of HFD on serum lipid, adipose tissue weight, and body weight [63, 65]. In a similar study, garlic (6 grams per 100 grams (6%)/day) could decrease LDL and TG levels in obese rats [71]. Kagawa et al. [63] showed that garlic oil (80 mg/kg, p.o.) could suppress gaining weight and WAT mass in the rats receiving a fat-rich diet through enhancing UCPI expression and increasing the oxidation of fat [63]. Lee et al. [72] indicated that the antiobesity impacts of garlic were exerted through AMPK activation, decrease in adipogenesis, and increase in thermogenesis [72]. Our findings consistent with these evidences indicated that the hepatic expression of PPAR*α*, CD36, and CTP-1beta decreased and SREBP-1c expression increased by HFD, whereas pretreatment or treatment with garlic could modulate the expression of these genes in liver tissue. Lai et al. [40] demonstrated that consuming different doses of garlic essential oil (25, 50, and 100 mg/kg) and the organosulfur components (DADS and diallyl disulfide) (10 and 20 mg/kg) could dose-dependently inhibit the inflammatory responses induced by the HFD [40]. The anti-HFD impacts of DADS and GEO were mediated by downregulation of sterol regulatory element that binds acetyl-CoA carboxylase, protein-1c, 3-hydroxy-3-methylglutaryl-coenzyme A reductase, and fatty acid synthase [40]. Moreover, methanolic extract of black garlic could normalize the expression of lipogenesis-related genes [73]. The findings indicated that garlic can potentially prevent liver damage caused by the HFD by normalizing the expression of genes involved in lipogenesis, fatty acid *β* oxidation, and fatty acid transport.

Clearly, the improvement of hepatic lipid metabolism in obesity by the consumption of medicinal plants is associated with the reduction of local and systemic inflammation and oxidative damage [3, 28, 42, 55]. Our results showed that testicular MDA increased and the activity of SOD and GPx declined in the rats with obesity which is consistent with other studies [3, 74]. Based on our findings, it may be due to decreased synthesis, increased degradation, or inactivation of antioxidant enzymes caused by high level of ROS production [8]. Nrf2 is a cytoprotective gene involved in regulating antioxidant response elements (AREs) [75]. It increases the expression of antioxidants and anticytotoxic genes like SOD, Heme oxygenase-1 (HO-1), CAT, NAD(P)H-quinone oxidoreductase 1 (NQO1), GST, and GPx [76, 77]. Specifically, increase in antioxidant mechanisms serves as the protective mechanisms for sperm in the testicular microenvironment and enhances spermatogenesis in the testes [13, 24, 28, 78]. In addition, previous studies have also shown that HFD and ethanol downregulated Nrf2 testicular expression and improving its expression are associated with enhancing testosterone production and its serum level [3, 28, 79]. Our results clearly showed that pretreatment and treatment with garlic enhance the expression of Nrf2 and antioxidant enzymes activity and decrease the level of MDA in the testicular tissue of obese rats. In line with our results, it has been demonstrated that garlic extract as an antioxidant reduced the toxic impacts of free radicals caused by testicular detorsion and torsion via its active compounds like diallyl sulfide (DAS), diallyl disulfide (DADS), diallyl thiosulfonate (allicin), diallyl trisulfide (DATS), S-allyl-cysteine (SAC), E/Z-ajoene, allylmethyl trisulfide, and S-allyl-cysteine sulfoxide (alliin) called organosulfurs [80, 81]. Their biological activities including the effects of reducing blood fat and cholesterol, antioxidant potential, and antimicrobial activity have been reported in many studies [82, 83]. Moreover, garlic extract could improve cognitive deficits and mitochondrial dysfunction of the brain in rats with obesity [70]. Moreover, Padiya et al. [84] showed that “cardiac oxidative stress is decreased by garlic through activation of pi3k/akt/nrf2-keap1 pathway in fructose-fed diabetic rat” [84]. The pungent parts of garlic are mainly moieties containing sulfur, while the two chemical groups including ALK (EN)-based cysteine sulfoxides (ACSOs) and flavonoids are good for health [85]. Our results showed that HFD could induce testicular inflammation by increasing the levels of TNF-*α* and IL-1*β* and the expression of NF-*κ*B, while pretreatment and treatment with garlic could inhibit inflammation induced by HFD. Obese individuals also experience chronic inflammation in various tissues via activating specific signaling pathways [86, 87]. There is evidence that TNF-*α* stimulates the activation of NF-*κ*B in an inflammatory response [88]. TNF-*α* induces cellular apoptosis through the extrinsic pathway, disturbs Sertoli cell junctions, and inhibits steroidogenesis in Leydig cells, as well as controls the size of the germ cell population in the seminiferous epithelium [89]. NF-*κ*B is also capable of regulating male germ cell apoptosis by activating caspase-8, which acts on the extrinsic apoptotic pathway [90]. In line with these results, our findings showed that in addition to the induction of inflammation and oxidative damage by the HFD in the testicular tissue, the rate of apoptosis also increased. The findings also indicated that the expression of BAX, Cas-3, and -9 genes increased and the expression of Bcl2 gene decreased in the testicular tissue of obese rats. Considering the extensive role of ROS and its abnormal levels in cells after long-term consumption of HFD, it can be considered that oxidative damage and inflammation have a significant role in inducing apoptosis in testicular tissue. Moreover, increased caspase-8 transcript levels in testes suggest the presence of a secondary apoptotic pathway, which is consistent with elevated levels of TNF-*β* and NF-*κ*B, two factors known to stimulate a secondary apoptotic pathway [90–92]. These results can well show the causes of tissue damage caused by HFD in obese rats, which was observed in the histological evaluation of the testes of obese rats. The findings also indicated that the expression of BAX, Cas-3, and -9 genes increased and the expression of Bcl2 gene decreased in the testicular tissue of obese rats, which is consistent with many past studies. However, the pretreatment and treatment of garlic in different doses in our study could attenuate the level of inflammatory parameters and the expression of NF-*κ*B gene and the expression of BAX, Cas-3, and -9 genes and increased the expression of Bcl2 gene in the testicular tissue of obese rats. Very few studies have investigated its antiapoptotic role. The active and key element of garlic extract is S-allyl cysteine which has antioxidant, anticancer, and antiliver toxicity activities [93, 94]. Another main garlic constituent is allicin which can exhibit antiapoptotic effects [95]. It is also proved that allicin prevents proapoptosis expression genes and decreases the cytochrome C level spread from mitochondria [95]. In line with our study, Lai et al. [40] showed that “consuming different doses of garlic essential oil (25, 50, or 100 mg/kg) could dose-dependently enhance antioxidant enzyme activities (SOD and GPx) and inhibit the inflammatory responses (TNF-*α* and IL-1*β*) in the liver in HFD-fed mice” [40]. These findings suggested that garlic or its derivatives can directly or indirectly, by inhibiting oxidative damage apoptosis induced by the HFD, activate the pathways of testosterone synthesis and spermatogenesis.

Our findings demonstrated that using a variety of doses of garlic as pretreatment and treatment improved the activity of the pituitary-gonadal axis and the expression genes that have a role in synthesis of testosterone, i.e., StAR, P450Scc, 17*β* HSD, and CYP19A1 in the testes tissue of HFD-fed rats. In line with these results, the findings of the histological analysis of the testicular tissue in the control and HFD groups (pre)treated with garlic indicated that the morphology of the seminiferous tubules was not changed and kept its normal cycle of spermatogenesis. However, morphological abnormalities including increased abnormal spermatogenesis and degeneration of sperm tubes are clearly evident in the testicular tissue of obese rats, which is consistent with what has been previously reported [3]. Oi et al. [96] reported that “using supplementation with 0.8 g/100 g garlic changes the hormones that play a role in the anabolism of protein through attenuating plasma corticosterone and improving testicular testosterone in rats receiving protein-rich diet”[96]. These researchers also showed that “LH concentration of plasma had a direct relationship with diallyldisulfide (a key sulfur-containing volatile compound) and IV administration of diallyldisulfide corresponded to absorption of garlic in blood following oral use of garlic” [96]. Memudu et al. [49] also showed the supportive role of garlic in a dose-dependent manner in regulating body weight and maintaining the integrity and function of the testis [49]. Our results in line with the results of this research showed that “the testis weight in subjects experiencing chronic and acute consumption of garlic increases, and a stronger increase in testis weight was observed in chronic administration of garlic” [49]. In fact, the increase in the weight of the testis and the histological properties of this tissue after consuming garlic show the improvement of the condition of its functional cells, i.e., cells that produce testosterone and the processes related to spermatogenesis. In these studies, it was found that garlic increases the level of testosterone in a dose-dependent manner. However, some studies show that raw garlic extract reduces testosterone serum levels. The difference in our results may be explained by the use of different administration doses along with different terms of exposure to garlic and different factors involved in the pathogenesis of the disease [97, 98]. Moreover, Rana et al. [99] also showed that “a key organosulfur compound in aged-garlic extract, S-allyl cysteine (SAC), enhances the testosterone level through activating the PKA pathway and can be a probable target for hypogonadism therapeutics” [99]. Garlic contains high amounts of zinc and selenium, and these substances along with diallyl sulfide have a regulatory role in testicular activities, especially the production of steroidogenic enzymes [100]. Moreover, in a similar study, Li et al. [28] showed that ginger can enhance reproductive dysfunction caused by ethanol through improving inhibiting oxidative stress, steroidogenesis, and inflammation [28]. However, it is possible that another mechanism is involved in the protective and therapeutic role of garlic. In this study, it has been shown that the expression of aromatase in the testicular tissue of obese rats is significantly high. However, pretreatment and treatment using a variety of garlic doses could bring its expression level in obese rats closer to its levels in the control group. Previous studies have well established that total and free testosterone levels are lower compared to nonobese men, and obese men have high levels of circulating estrogen, which results from the conversion of androgens to estrogen by the aromatase enzyme [101]. In addition, increased adipose tissue is related to overexpression of TNF-*α* and IL-6, which also improves production of aromatase, acting in an autocrine or paracrine fashion [101–103]. In this study, it was shown that the level of insulin could improve after (pre)treated rats with a variety of doses of garlic in male rats with obesity. It was well demonstrated that insulin and leptin are mediators and modulators of the hypothalamic-pituitary-testicular axis that help regulate male reproductive potential [104]. In line with our study “many research works have shown that using moderate amounts of garlic (one clove at least or equivalent daily) can regulate blood sugar levels and improve insulin sensitivity” [105, 106]. As the findings showed, we can conclude that using garlic in a dose-dependent activity causes an increase in serum and tissue testosterone levels by increasing the expression of testosterone synthesis, i.e., StAR, P450Scc, and 17*β*HSD in the testis tissues. These effects can be inhibiting local or systemic inflammation and inhibiting apoptosis and oxidative damage in the testicular tissue, improving the activity of the pituitary-gonadal axis and improving the metabolic state of glucose or lipids in the liver tissue. These findings can provide a new window for the therapeutic use of garlic powder or its products as a modifier of metabolic and reproductive disorders in obese people. An achievement is well demonstrated by many studies related to alternative and complementary medicine [3, 24, 25, 28, 48, 107]. One of the limitations we faced in this study was the long time it took to induce obesity. Therefore, it is suggested that obesity models related to the use of drugs, including corticosteroids, are also used. In addition, it is suggested that the receptors of sex hormones are also evaluated in the reproductive organs in order to discover the cellular effects.

## Figures and Tables

**Table 1:** The primer sequences used in this study.

Genes	Sequences	PCR products
NF-*κ*B	Forward 5′- GCACCAAGACCGAAGCAAT-3′	143
	Reverse 5′- CGTAACCGCGTAGTCGAAGA -3′	

SREBP-1c	Forward 5-TCCTGGAGCGAGCATTGAA -3	119
	Reverse 5′- GACAGCGTCAGAACAGCTATTTAG -3′	

CPT-1beta	Forward 5′- TCAACCTCGGACCCAAATTG-3′	101
	Reverse 5′- GCCCCGCAGGTAGATATATTC-3′	

Nrf-2	Forward5′-AAAGACAAACATTCAAGCCGATTAG-3′	141
	Reverse 5′- TTGCTCCTTGGACATCATTTCAT -3′	

CD36	Forward5′- GATGTGGAACCCATAACTGGA -3′	166
	Reverse 5′- CTTTCTCATCGCCAATGGTC -3′	

FAS	Forward5′- TTGATGATTCAGGGAACGGG-3′	114
	Reverse 5′- TGTTCGTCCCGGCATTCA-3′	

CYP19A1	Forward5′- CACATCCTCAATACCAGGTCC-3′	143
	Reverse 5′- CAGAGATCCAGACTCGCATG-3′	

P450scc	Forward5′- AACAACTACTTCCGCAGCCT -3′	170
	Reverse 5′- CGGTAGAACAATGAGCTGGA -3′	

StAR	Forward 5′- CTGAGGCAACAGGCTGTGAT-3′	114
	Reverse 5′-AGCCGAGAACCGAGTAGAGAG-3′	

17*β*HSD	Forward 5′-CGCCTCAGGAACCTCGTCT-3′	62
	Reverse 5′- GCTGGCGCAATAAACGTCA-3′	

PPAR*α*	Forward5′- ACGATGCTGTCCTCCTTGATG -3′	407
	Reverse 5′- GCGTCTGACTCGGTCTTCTTG-3′	

Beta-actin	Forward5′-TTGCTGACAGGATGCAGAAGG-3′	134
	Reverse 5′- CTGGAAGGTGGACAGTGAGGC-3′	

**Table 2:** The mean ± SD of final body weight, liver weight, testis weight, and tissue index in different groups.

	Final weights (g)	Liver weights (g)	Liver indexes (%)	Testis weights (g)	Testis indexes (%)
Control	245 ± 23a	12.24 ± 4a	4.89	9.36 ± 1a	3.67
Garlic 50	241 ± 21a	12.36 ± 3a	4.93	8.89 ± 1a	3.46
Garlic 100	230 ± 24ad	12.45 ± 2a	5.21	9.16 ± 2a	3.91
Obesity	324 ± 31b	15.14 ± 3b	4.32	10.94 ± 2b	3.08
Garlic50-obesity	264 ± 25c	13.48 ± 2c	6.06	10.14 ± 2b	3.78
Garlic100-obesity	251 ± 24a	11.98 ± 4a	8.76	9.94 ± 2b	3.58
Obesity-garlic50	234 ± 26ad	13.61 ± 2c	8.97	10.16 ± 2b	4.27
Obesity-garlic100	224 ± 23d	12.18 ± 3a	9.37	11.14 ± 2b	4.91

^*∗*^Mismatched lowercase letters indicate a significant difference (*p* < 0.05).

**Table 3:** The mean ± SD of fasting blood glucose (mg/dl) and insulin (*μ*U/l) levels in different groups^*∗*^.

Groups	Fasting blood sugar (mg/dL)			Insulin (*μ*U/L)		HOMA-IR	
	1^st^ week	12^th^ week	24^th^ week	12^th^ week	24^th^ week	12^th^ week	24^th^ week
Control	97 ± 11.4a	98 ± 13.4a	103 ± 12.5a	11 ± 2.11	12 ± 1.30a	1.45a	1.6a
Garlic 50	98 ± 11.4a	89 ± 16.3a	—	11 ± 2.45a	—	1.42a	—
Garlic 100	97 ± 14.4a	90 ± 12.4a	—	10 ± 2.31a	—	1.29a	—
Obesity	95 ± 13.4a	254 ± 26.1b	263 ± 10.6b	14.8 ± 2.12b	15.9 ± 1.62b	2.52b	2.78b
Garlic50-obesity	96 ± 11.0a	134 ± 13.1c	—	13 ± 2.10c	—	1.83c	—
Garlic100-obesity	98 ± 13.0a	120 ± 11.1d	—	12 ± 2.03d	—	1.66a	—
Obesity-garlic50	96 ± 10.4a	265 ± 12.3e	158 ± 11.5cA	15.3 ± 2.1e	13.7 ± 1.74cA	2.7b	2cA
Obesity-garlic100	94 ± 11.4a	259 ± 10.1e	134 ± 12.8dA	15.2 ± 2.41e	12.4 ± 1.25aA	2.62b	1.75aA

Lowercase letters show a significant difference at 12^th^ week and 24^th^ week between the different groups. Mismatched uppercase letters indicate a significant difference between the 12^th^ and 24^th^ weeks (*p* < 0.05).

**Table 4:** The mean ± SD serum levels of glucose, total cholesterol (TC), triglyceride (TG), low-density cholesterol (LDL), and high-density cholesterol (HDL) in different groups^*∗*^.

Groups	Glucose (mg/dL)	TC (mg/dL)	TG (mg/dL)	HDL (mg/dL)	LDL (mg/dL)
Control	103 ± 14.1a	76.61 ± 11.4a	94.3 ± 6.8a	39.8 ± 9.4a	47.8 ± 5.6a
Garlic 50	89.1 ± 12.4b	74.47 ± 8.6a	91.4 ± 10.2a	36.9 ± 8.6a	45.4 ± 4.5a
Garlic 100	90.4 ± 21.4b	72.14 ± 7.84a	84.6 ± 8.9b	34.3 ± 7.8a	44.9 ± 6.4a
Obesity	263.1 ± 21.4c	137.9 ± 9.8b	154.5 ± 6.8c	76.7 ± 8.6b	68.1 ± 3.6b
Garlic50-obesity	134.7 ± 2.14d	109.4 ± 8.7c	121.9 ± 9.8d	56.7 ± 9.8c	58.5 ± 2.3c
Garlic100-obesity	120 ± 11.04e	91.9 ± 8.4d	108.7 ± 11.7e	44.8 ± 8.7d	51.4 ± 6.4d
Obesity-garlic50	158.8 ± 14.6f	114.7 ± 9.1e	131.8 ± 10.7d	43.9 ± 8.6d	54.8 ± 6.8d
Obesity-garlic100	134.6 ± 12.6d	84.7 ± 8.1f	110 ± 11.7e	37.3 ± 9.7a	48.7 ± 7.8a

^*∗*^Mismatched lowercase letters indicate a significant difference, *p* < 0.05.

**Table 5:** The mean ± SD number of spermatogonia, spermatocytes, spermatids, Leydig cells, Sertoli cells, and Johnsen score in the different groups^*∗*^.

Groups	Spermatogonia	Spermatocytes	Spermatids	Leydig cells	Sertoli cells	Johnsen score
Control	64 ± 11a	76.1 ± 11.4a	64.3 ± 6.8a	17.8 ± 1.4a	7.8 ± 1.6a	9.25 ± 0.12a
Garlic 50	65 ± 12a	74.7 ± 8.6a	71.4 ± 10.2a	18.9 ± 1.6a	8.4 ± 1.5a	9.44 ± 0.22a
Garlic 100	69 ± 11a	72.4 ± 7.84a	74.6 ± 8.9b	19.3 ± 2.8a	8.9 ± 1.4a	9.58 ± 1.21a
Obesity	48 ± 14b	57.9 ± 9.8b	48.5 ± 6.8c	15.7 ± 2.6b	5.1 ± 1.6b	7.94 ± 0.1b
Garlic50-obesity	56 ± 10c	63 ± 8.7c	63.9 ± 9.8a	17.7 ± 1.8a	7.1 ± 1.3c	9.12 ± 0.4a
Garlic100-obesity	63 ± 11a	69 ± 8.4c	68.7 ± 11.7a	18 ± 1.7a	7.4 ± 1.4c	9.29 ± 0.1a
Obesity-garlic50	52 ± 14bc	64.7 ± 9.1c	59.8 ± 10.7d	16.9 ± 1.6c	6.8 ± 1.8d	9.09 ± 0.2a
Obesity-garlic100	58 ± 1c	67 ± 8.1c	65 ± 11.7a	17.3 ± 2.7a	7.7 ± 1.8c	9.34 ± 0.4a

^*∗*^Mismatched lowercase letters indicate a significant difference. *p* < 0.05.

## Data Availability

The data used to support the findings of this study are available from the corresponding author upon request.
